# Adverse childhood experiences and pain incidence and multisite trajectories among middle-aged and older Chinese adults

**DOI:** 10.1097/PR9.0000000000001389

**Published:** 2026-01-09

**Authors:** Jiajia Li, Heming Pei, Guilan Xie, Gong Chen, Lijun Pei

**Affiliations:** a Institute of Population Research, Peking University, Beijing, China; b Butler Columbia Aging Center, Columbia University Mailman School of Public Health, New York, NY, USA; c Department of Epidemiology, Columbia University Mailman School of Public Health, New York, NY, USA

**Keywords:** Pain, ACEs, Cox model, Trajectory analysis, Depressive symptoms

## Abstract

**Introduction:**

Pain is prevalent among middle-aged and older adults, and adverse childhood experiences (ACEs) may contribute to its development; however, mechanisms linking ACEs to pain remain unclear.

**Objectives:**

This study investigates the association between ACEs and incident pain and multisite pain trajectories, focusing on mediation by depressive symptoms and social isolation.

**Methods:**

We used China Health and Retirement Longitudinal Study data (2011–2020). We analyzed 2 samples: an incidence cohort of pain-free at baseline (n = 9,252) and a trajectory cohort with repeated pain measures (n = 9,935). Pain was assessed at each wave by self-reported presence and multisite pain trajectories. Adverse childhood experiences were measured using a cumulative index of 13 adversities. Cox proportional hazards models, group-based trajectory modeling, and multinomial logistic models estimated associations with incident pain and trajectory group membership. Multiple mediation analyses quantified indirect effects of depressive symptoms and social isolation.

**Results:**

We identified 3 multisite pain trajectories: minimal multisite (46.33%), increasing multisite (38.93%), and high multisite (14.74%). Higher ACEs scores (≥4) were associated with increased risk of incident pain (hazard ratio = 1.20, 95% confidence interval [CI]: 1.10–1.32) and membership in high multisite trajectories (odds ratio = 3.97, 95% CI: 3.05–5.17). In parallel mediation models, depressive symptoms mediated up to 28.25% of the ACEs–pain association for high multisite trajectories, whereas social isolation showed minimal effects.

**Conclusion:**

Adverse childhood experiences were linked to incident and multisite pain, largely through depressive symptoms. Targeting psychological health may mitigate the impact of ACEs on pain outcomes, suggesting a need for interventions addressing early-life exposures and mental health interventions in aging populations.

## Abstract 2

Supplemental Digital Content is Available in the Text.

Adverse childhood experiences associated with body pain incidence and multisite trajectory in older adults, primarily mediated by depressive symptoms. Targeting psychological health may mitigate long-term pain consequences.

## 1. Introduction

Pain is common and debilitating among middle-aged and older adults, impairing physical, emotional, and social well-being.^8^ Pain phenotypes such as incident (new-onset) pain and multisite pain are consequential for health in later life.^29,39^ Incident pain predicts subsequent declines in healthy aging,^39^ whereas multisite pain is associated with poorer disability trajectories and increased fracture risk.^22,30^ Consistent with the biopsychosocial model,^28^ pain arises from the complex interaction of biological, psychological, and social factors, making its management particularly challenging and heightening its burden on individuals, families, and health care systems.^34^ Among psychological factors, depression is an important contributor, as it worsens pain outcomes and increases disability risk.^16^ In addition, the accumulation of adversities throughout the life course may increase the risk and severity of pain,^2^ making it a significant public health issue.

Adverse childhood experiences (ACEs), including abuse, neglect, and household dysfunction, are widely recognized as significant determinants of health outcomes,^11,14^ including pain in adulthood. Adverse childhood experiences can disrupt neurobiological maturation, stress regulation, and cognitive development, with greater impact during stress-sensitive periods.^12^ Previous studies have shown that ACEs are associated with chronic pain and pain-related disability in adulthood,^4^ with a dose–response relationship observed.^26^ Moreover, evidence indicates that the association between ACEs and later life pain is cumulative, with higher ACEs experiences associated with increased risk of pain across the life course.^14,26^ These cumulative effects, like early psychosocial and biological dysregulation, persist into later life and interact with age-related conditions such as arthritis^27^ and pain in middle-aged to older adults.^23,24^

Despite the established link between ACEs and pain, the psychosocial mechanisms that mediate this relationship remain insufficiently explored. Guided by the biopsychosocial model of chronic pain^28^ and life-course cumulative adversity perspectives, we prespecified depressive symptoms (psychological pathway) and social isolation (social pathway) as the mediators of interest. Depressive symptoms, which are highly prevalent among individuals with a history of ACEs,^18^ may mediate this relationship by altering pain perception, reducing resilience to stress, and impairing emotional regulation,^21,35^ thereby increasing risks of pain onset and multisite development. Social isolation, a common issue among older adults experiencing ACEs,^3^ captures objective/structural disconnectedness and is theorized to affect pain via weakened stress-buffering, may also intensify the impact of ACEs by limiting access to emotional and practical support systems that are essential for coping with pain.^36^ However, few studies have comprehensively examined how these psychosocial factors jointly mediate the relationship between ACEs and incident and multisite pain, particularly in the context of aging populations such as China.

This study aims to investigate the relationship between ACEs and pain in a nationally representative cohort of middle-aged and older adults in China. The primary objective is to explore how ACEs are associated with the incident pain and membership in trajectories characterized by an increasing or persistently high number of painful body sites over time. We hypothesize that higher ACEs scores will be significantly associated with an increased risk of incident pain and multisite pain trajectories. The secondary objective is to examine the mediating roles of depressive symptoms and social isolation in this relationship. Specifically, we hypothesize that depressive symptoms will mediate the association between ACEs and pain, representing parallel psychosocial pathways. We did not include anxiety or other perceived social constructs due to the unavailability of consistently measured, validated data across waves. By investigating these psychosocial pathways, the findings are expected to inform the design of targeted mental health interventions for older adults, particularly those with a history of childhood adversity, aimed at mitigating depression and reducing the burden of pain.

## 2. Method

### 2.1. Data and samples

We used data from the China Health and Retirement Longitudinal Study (CHARLS), a nationally representative longitudinal survey of Chinese adults aged 45 years and older. China Health and Retirement Longitudinal Study collects high-quality data through a multistage probability sampling design, which includes detailed information on demographics, socioeconomic status, health outcomes, and life history. Detailed information on the survey design has been published previously.^42^ For this study, we used the 5 core waves conducted in 2011 (Wave 1, baseline), 2013 (Wave 2), 2015 (Wave 3), 2018 (Wave 4), 2020 (Wave 5), and the 2014 Life History Survey. We employed 2 analytical samples: one to investigate incident pain (n = 9,252, excluding individuals who already reported body pain at baseline) and another to examine pain trajectories over time (n = 9,935, requiring participants with valid pain measures across multiple waves). Figure 1 illustrates the exclusion criteria for each analytical sample.

**Figure 1.**
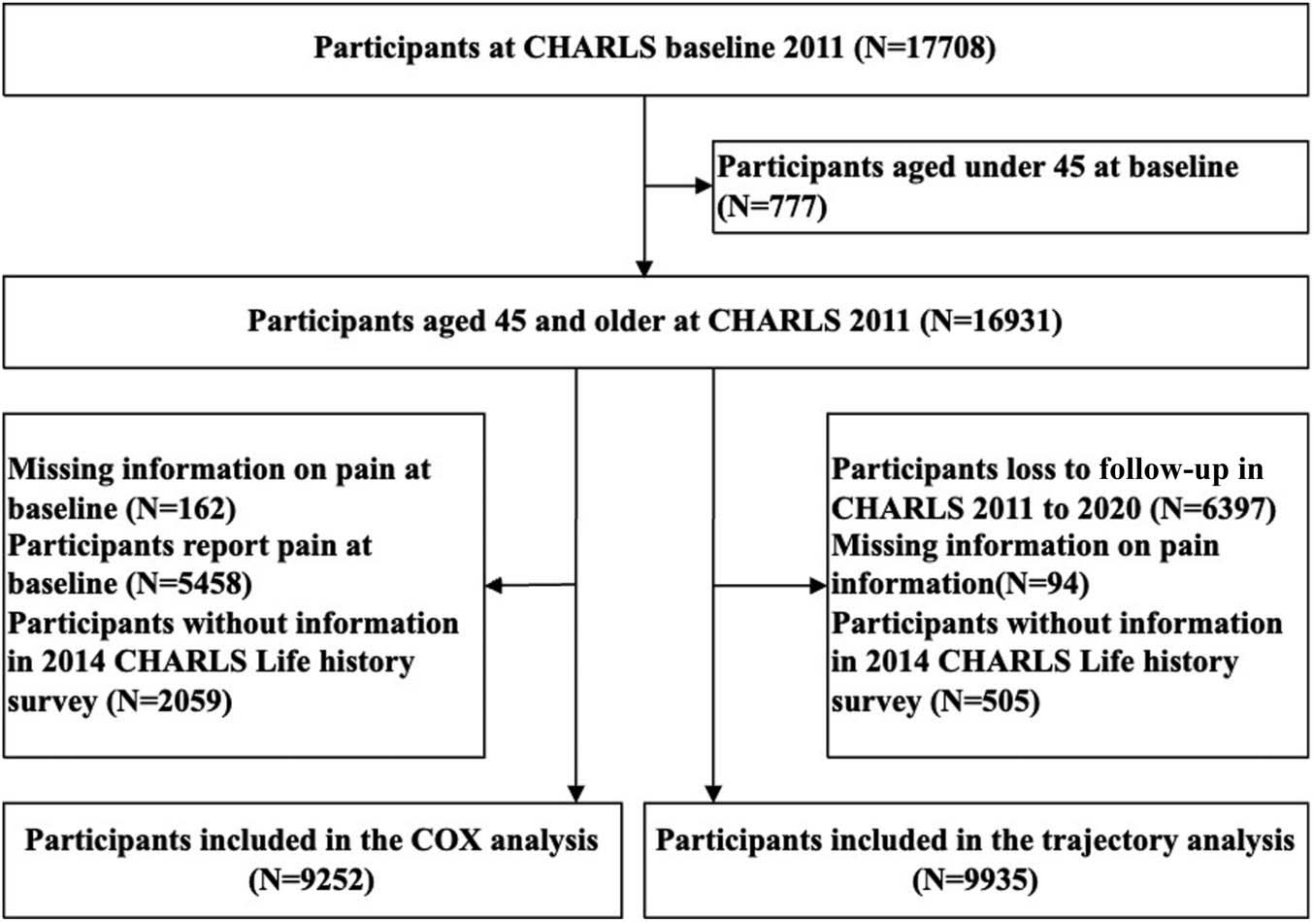
Sample selection process. This flowchart illustrates the participant selection process for 2 analyses in the study. Starting from the CHARLS 2011 baseline sample (N = 17,708), individuals under 45 were excluded. For the Cox analysis, participants with pain at baseline, missing pain data, or missing 2014 Life History data were excluded, yielding a final sample of 9,252. For the trajectory analysis, exclusions included participants lost to follow-up, missing pain data, and missing 2014 data, resulting in a sample of 9,935. This approach allows for separate analyses of pain incidence and pain trajectory patterns. CHARLS, China Health and Retirement Longitudinal Study.

### 2.2. Outcomes

Pain was assessed at each wave of the CHARLS. Participants were initially asked, “Are you often troubled with any body pains?” and if they responded affirmatively, they were then asked to identify all affected body sites from a predefined list of 15 locations, including head, shoulders, arms, wrists, fingers, chest, stomach, back, waist, buttocks, legs, knees, ankles, toes, and neck. We defined 2 outcomes: (1) incident (new-onset) pain, the first report of pain after a pain-free baseline, and (2) multisite pain distribution over time, operationalized as the number of painful body sites (0–15) at each wave and its trajectory across follow-up. The site count indexes the distribution/widespreadness of pain rather than intensity or mechanism and has been used in prior studies to characterize pain distribution over time.^23^

### 2.3. Exposures

Adverse childhood experiences were assessed using data from the 2014 life history survey of the CHARLS. Adverse childhood experiences were measured through 13 items capturing various forms of childhood adversity, including parental death or separation, physical abuse, emotional neglect, and family dysfunction. Each item was defined as present (1) or absent (0), and the scores were summed to create a cumulative ACEs index ranging from 0 to 13, with higher scores indicating greater exposure to childhood adversity. The definition and measurement of ACEs were based on established frameworks and methodologies from previous studies.^1,11^ For this study, ACEs scores were categorized into 5 levels: 0 (no exposure), 1, 2, 3, and ≥4 (high exposure). This classification is consistent with the thresholds employed in previous research^19^ and facilitates the examination of the dose–response relationship between ACEs and pain outcomes.

### 2.4. Mediators

Two psychosocial mediators were examined: depressive symptoms and social isolation, both of which were measured at each core CHARLS wave: 2011, 2013, 2015, 2018, and 2020. Anxiety and loneliness were not included due to the absence of validated, repeatedly measured items in CHARLS; the only proximate psychiatric indicator is a single binary doctor-diagnosed item, which was included in the chronic disease burden covariate.

Depressive symptoms were assessed using the 10-item version of the Center for Epidemiologic Studies Depression Scale (CES-D 10), a well-validated instrument widely employed in population-based research. Each item was rated on a 4-point scale, and the total score was standardized as a Z-score for direct comparability, with higher scores reflecting greater severity of depressive symptoms. The CES-D 10 has been shown to be a reliable measure for assessing depressive symptomatology in older adults and has been used in numerous large-scale studies to examine the impact of depression on health outcomes.^15^

Social isolation was measured through a composite index based on multiple objective indicators of social isolation, as informed by prior research.^40^ The index included marital status, whether any child coresides with the respondent, whether a child lives nearby, frequency of weekly contact with parents or parents-in-law in person, frequency of weekly contact with children in person, by phone, mail, or email, and participation in social groups. The social isolation index ranged from 0 to 11, with higher scores indicating greater social isolation and reduced levels of social engagement. To enhance comparability across mediators and waves, the social isolation index was also z-standardized by wave before analysis. The items and coding are listed in Supplementary Table S1, http://links.lww.com/PR9/A324.

### 2.5. Covariates

We adjusted for several covariates, including age (45–49, 50–54, 55–59, 60–64, 65–69, 70–74, and ≥75 years), sex (male or female), marital status (married or unmarried), region (urban, rural), education (illiterate, able to read, elementary, middle school, high school or above), smoking status (current vs non), drinking status (current vs noncurrent), annual per capita household expenditure (in quartiles), and chronic disease burden. Chronic disease burden was defined as the simple sum (range 0–14) of doctor-diagnosed conditions: hypertension, dyslipidemia, diabetes or high blood sugar, cancer or malignant tumor, chronic lung disease, liver disease, heart problems, stroke, kidney disease, stomach or other digestive disease, emotional, nervous, or psychiatric problems, memory-related disease, arthritis or rheumatism, and asthma. Each condition was coded 1 if the respondent reported having been told by a doctor that they had the condition and 0 otherwise; the resulting count was categorized as 0, 1, 2, 3, or ≥4 in analyses.

### 2.6. Statistical analysis

We conducted descriptive analyses to summarize the baseline characteristics of participants across levels of ACEs. Continuous variables were presented as means with standard deviations, and categorical variables were reported as frequencies with percentages. We used the Kruskal–Wallis test for continuous variables to compare characteristics across ACEs levels and Pearson χ^2^ test for categorical variables.

#### 2.6.1. Incidence of pain

We applied Cox proportional hazards models to estimate hazard ratios (HRs) and 95% confidence intervals (CIs) for body pain onset, adjusting for covariates. Time-to-event was calculated from baseline (2011) to first report of body pain or end of follow-up (2020).

#### 2.6.2. Pain trajectories

We also examined trajectories in the number of painful body sites over time using group-based trajectory modeling, modeling the count of painful body sites from 2011 to 2020 with a zero-inflated Poisson distribution to accommodate excess zeros. Trajectory solutions with 2 to 5 classes were estimated. Model selection prioritized a Bayesian Information Criterion “elbow” for parsimony together with standard classification diagnostics: log-likelihood, Akaike Information Criterion, Bayesian Information Criterion, and cross-validation error.^25^ Participants were assigned to trajectory groups based on their maximum posterior probabilities. Full fit and classification diagnostics for 2 to 5 class solutions are shown in Supplementary Table S2, http://links.lww.com/PR9/A324. We then used multinomial logistic regression to assess the association between ACEs and trajectory groups, reporting results as odds ratios (ORs) with 95% CIs.

#### 2.6.3. Mediation analyses

We conducted mediation analyses to examine the indirect effects of depressive symptoms and social isolation on the relationship between ACEs and pain outcomes. To preserve temporal ordering in the incidence analysis, mediators were entered as time-updated, one-wave-lagged covariates. For the trajectory analysis, mediators were specified as baseline values to predict subsequent trajectory group membership.

We estimated parallel indirect effects using the product-of-coefficients approach implemented in the multiple mediation analysis (MMA),^41^ with bias-corrected bootstrap 95% CIs based on 1,000 resamples. We estimated the total, direct, and indirect effects, calculating the relative contributions of each mediator to the indirect effects as percentages of the total effect. The MMA models multiple mediators in parallel, allows correlation between them, and does not assume a causal order. The mediation analyses were conducted based on 2 different models: the Cox proportional hazards model for pain incidence and the multinomial logistic regression model for pain trajectories. These models allow us to capture both the onset and the distributional change of pain, respectively. The full regression-based parallel mediation framework and its temporal ordering are depicted in Supplementary Figure S1, http://links.lww.com/PR9/A324.

We also employed the multivariate imputation by chained equations (MICE) method with a random forest algorithm to handle missing data in covariates,^5,33^ restricting imputation to covariates only. We summarized missingness with heatmaps and tabulations (Supplementary Figures S2–S3, http://links.lww.com/PR9/A324; Tables S3–S4, http://links.lww.com/PR9/A324), and Little's Missing Completely at Random (MCAR) tests rejected MCAR in both analytic samples (*P* < 0.001), supporting a Missing at Random (MAR) working assumption while acknowledging Missing Not at Random (MNAR) cannot be ruled out. Imputations were run separately for the incidence and trajectory cohorts. Sensitivity analysis: Baseline differences by follow-up status and trajectory data completeness are summarized in Supplementary Tables S5–S6, http://links.lww.com/PR9/A324. To assess potential bias from differential attrition, we applied inverse-probability-of-attrition weighting (IPW) using baseline covariates. All statistical analyses were performed using R version 4.4.1,^31^ and statistical significance was defined as a two-tailed *P*-value <0.05.

## 3. Results

### 3.1. Baseline characteristics of study participants

Table 1 presents the baseline characteristics of participants, stratified by levels of ACEs. Participants with higher ACEs scores were generally younger, more likely to be male, and had lower levels of educational attainment. Rural residency was more prevalent among participants with higher ACEs, as were behaviors such as current smoking and drinking. Higher ACEs levels were also associated with a greater burden of chronic diseases and elevated levels of social isolation and depressive symptoms.

**Table 1:** Baseline characteristics of participants, by adverse childhood experiences level (n = 9,252).

Variable	ACEs levels					*P*
	0 (n = 1,138)	1 (n = 2,136)	2 (n = 2,357)	3 (n = 1,727)	≥4 (n = 1,894)	
Age (y)						<0.001
45–49	281 (24.69)	494 (23.13)	511 (21.68)	334 (19.34)	377 (19.90)	
50–54	197 (17.31)	337 (15.78)	364 (15.44)	283 (16.39)	275 (14.52)	
55–59	212 (18.63)	466 (21.82)	474 (20.11)	383 (22.18)	437 (23.07)	
60–64	160 (14.06)	349 (16.34)	385 (16.33)	299 (17.31)	335 (17.69)	
65–69	92 (8.08)	197 (9.22)	293 (12.43)	183 (10.60)	210 (11.09)	
70–74	79 (6.94)	156 (7.30)	183 (7.76)	136 (7.87)	136 (7.18)	
≥75	117 (10.28)	137 (6.41)	147 (6.24)	109 (6.31)	124 (6.55)	
Sex						<0.001
Female	584 (51.32)	1,090 (51.03)	1,096 (46.50)	762 (44.12)	820 (43.29)	
Male	554 (48.68)	1,046 (48.97)	1,261 (53.50)	965 (55.88)	1,074 (56.71)	
Marital status						0.200
Married	931 (81.81)	1,783 (83.47)	1,962 (83.24)	1,418 (82.11)	1,531 (80.83)	
Unmarried	207 (18.19)	353 (16.53)	395 (16.76)	309 (17.89)	363 (19.17)	
Education level						<0.001
Illiterate	260 (22.85)	519 (24.30)	562 (23.84)	456 (26.40)	483 (25.50)	
Can read	133 (11.69)	323 (15.12)	416 (17.65)	290 (16.79)	413 (21.81)	
Elementary school	226 (19.86)	439 (20.55)	530 (22.49)	400 (23.16)	420 (22.18)	
Middle school	305 (26.80)	517 (24.20)	512 (21.72)	392 (22.70)	368 (19.43)	
High school and more	214 (18.80)	338 (15.82)	337 (14.30)	189 (10.94)	210 (11.09)	
Region						<0.001
Urban	530 (46.57)	886 (41.48)	951 (40.35)	674 (39.03)	696 (36.75)	
Rural	608 (53.43)	1,250 (58.52)	1,406 (59.65)	1,053 (60.97)	1,198 (63.25)	
Smoke now						<0.001
No	808 (71.00)	1,514 (70.88)	1,596 (67.71)	1,088 (63.00)	1,229 (64.89)	
Yes	330 (29.00)	622 (29.12)	761 (32.29)	639 (37.00)	665 (35.11)	
Drink now						<0.001
No	774 (68.01)	1,427 (66.81)	1,526 (64.74)	1,033 (59.81)	1,172 (61.88)	
Yes	364 (31.99)	709 (33.19)	831 (35.26)	694 (40.19)	722 (38.12)	
Annual per capita household expenditure level						0.200
Q1	285 (25.04)	529 (24.77)	596 (25.29)	440 (25.48)	468 (24.71)	
Q2	236 (20.74)	528 (24.72)	577 (24.48)	436 (25.25)	498 (26.29)	
Q3	303 (26.63)	547 (25.61)	617 (26.18)	444 (25.71)	461 (24.34)	
Q4	314 (27.59)	532 (24.91)	567 (24.06)	407 (23.57)	467 (24.66)	
Chronic disease						<0.001
0	492 (43.23)	872 (40.82)	918 (38.95)	645 (37.35)	662 (34.95)	
1	332 (29.17)	648 (30.34)	752 (31.90)	542 (31.38)	608 (32.10)	
2	200 (17.57)	382 (17.88)	407 (17.27)	323 (18.70)	344 (18.16)	
3	66 (5.80)	144 (6.74)	169 (7.17)	125 (7.24)	160 (8.45)	
≥4	48 (4.22)	90 (4.21)	111 (4.71)	92 (5.33)	120 (6.34)	
Social isolation(z)	−0.09 (0.97)	−0.03 (1.03)	−0.02 (0.98)	0.05 (1.02)	0.07 (0.99)	<0.001
Depression symptoms(z)	−0.37 (0.81)	−0.38 (0.79)	−0.31 (0.82)	−0.23 (0.85)	−0.12 (0.88)	<0.001

Data are presented as n (%) for categorical variables and mean (SD) for continuous variables. Pearson χ^2^ test was used for categorical variables, and the Kruskal–Wallis rank sum test was used for continuous variables. ACEs variables are based on the 2014 survey, with higher scores indicating greater adversity. Depression symptoms are represented as a Z-score of the CES-D 10 scale. Chronic disease includes 14 conditions defined in the paper. Annual per capita household expenditure is categorized by quartiles, from Q1 (lowest) to Q4 (highest).

ACEs, adverse childhood experiences.

### 3.2. Association between adverse childhood experiences and pain incidence risk and trajectory

The left panel of Table 2 shows the association between ACEs and pain incidence. A significant dose–response relationship was observed, with increasing ACEs scores associated with a higher risk of pain incidence. Compared with those with no ACEs, participants with an ACEs score of 3 and 4 or more showed an increased risk of pain incidence (HR = 1.11; 95% CI: 1.01–1.22; HR = 1.20; 95% CI: 1.10–1.32, respectively), after adjusting for covariates.

**Table 2:** Association between adverse childhood experiences and pain incidence and multisite trajectories.

Variables	Model 1			Model 2	
ACEs score	No. of participants	No. of events	HR (95% CI)	Increasing multisite vs. minimal multisite	High multisite vs. minimal multisite
				OR (95% CI)	OR (95% CI)
0	1138	683	1.00	1.00	1.00
1	2136	1385	0.99 (0.90–1.09)	1.18 (1.00–1.40)	1.38 (1.04–1.82)*
2	2357	1566	1.04 (0.95–1.14)	1.34 (1.14–1.58)*	1.71 (1.31–2.24)*
3	1727	1262	1.11 (1.01–1.22)*	1.80 (1.52–2.14)*	2.65 (2.02–3.48)*
≥4	1894	1435	1.20 (1.10–1.32)*	2.22 (1.88–2.63)*	3.97 (3.05–5.17)*

Model 1 is a Cox proportional hazards model examining the association between ACEs and pain incidence risk, whereas model 2 is a multinomial logistic model assessing the association between ACEs and multisite pain trajectory membership. Both models are adjusted for age, sex, marital status, region, education, smoking, drinking, annual per capita household expenditure level, and chronic diseases. Hazard ratios (HR) with 95% confidence intervals (CI) are presented for model 1, whereas odds ratios (OR) with 95% CI are presented for model 2.

* 95% CI excludes 1.00 (2-sided *P* < 0.05).

ACEs, adverse childhood experiences; CI, confidence interval; HR, hazard ratio; OR, odds ratio.

Group-based trajectory modeling (Fig. 2) identified 3 distinct pain trajectories from 2011 to 2020: minimal multisite (46.33%), increasing multisite (38.93%), and high multisite (14.74%). The minimal multisite group consistently reported few or no painful body sites over time. The increasing multisite group exhibited a gradual increase in the number of painful body sites, whereas the high multisite group maintained persistently high counts of painful body sites throughout the study period, reflecting substantial heterogeneity in multisite pain distribution over time.

**Figure 2.**
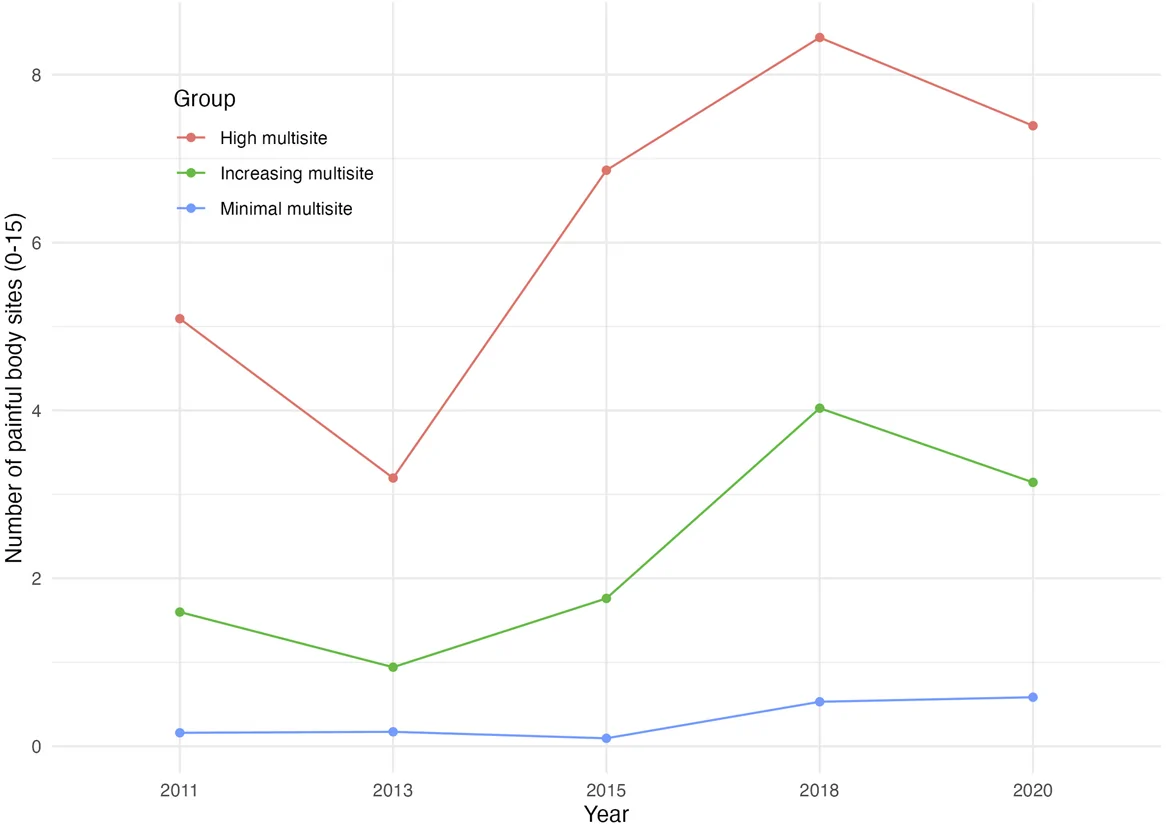
Estimated development trajectories of Pain sites numbers from 2011 to 2020. Trajectories were estimated using group-based trajectory model (GBTM) with a zero-inflated Poisson specification. Model selection was guided by Bayesian Information Criterion (BIC), and individuals were assigned to a trajectory group based on their maximum posterior probability. This model identified 3 distinct trajectory groups based on longitudinal pain patterns: minimal multisite (46.33%), increasing multisite (38.93%), and high multisite (14.74%).

The right panel of Table 2 shows the associations between ACEs and trajectory group membership of pain. Compared with the minimal multisite group, participants with higher ACEs scores were more likely to belong to the increasing multisite and high multisite groups. For the increasing multisite trajectory group, participants with ACEs scores of 2, 3, and 4 or more demonstrated progressively higher risk of being in the group (OR = 1.34; 95% CI: 1.14–1.58, OR = 1.80; 95% CI: 1.52–2.14, and OR = 2.22; 95% CI: 1.88–2.63, respectively). For the high multisite trajectory group, participants with an ACEs score of 1, 2, 3, and 4 or more demonstrated progressively higher risk of being in the trajectory (OR = 1.38; 95% CI: 1.04–1.82; OR = 1.71; 95% CI: 1.31–2.24; OR = 2.65; 95% CI: 2.02–3.48; and OR = 3.97; 95% CI: 3.05–5.17, respectively). These results highlight a strong and consistent relationship between higher levels of childhood adversity and the likelihood of experiencing persistently high multisite pain over the follow-up period. The IPW-adjusted estimates for both the incidence (Cox) and trajectory (multinomial) models were similar to the primary results (Supplementary Table S7, http://links.lww.com/PR9/A324).

### 3.3. Mediation effects of depressive symptoms and social isolation

Table 3 presents the mediation effects of depressive symptoms and social isolation on the association between ACEs and pain outcomes.

**Table 3:** Mediation analysis of depression and social isolation on the association between adverse childhood experiences and pain incidence risk and trajectories.

Variables	ACEs level (Ref. = 0)			
	1	2	3	≥4
Incident pain (Cox model)				
Total effect^a^	0.99 (0.95–1.04)	1.04 (1.01–1.09)*	1.13 (1.08–1.19)*	1.21 (1.14–1.25)*
Direct effect	0.99 (0.94–1.04)	1.04 (1.00–1.06)	1.11 (1.05–1.18)*	1.17 (1.11–1.21)*
Indirect effect	1.00 (0.99–1.01)	1.01 (1.00–1.02)	1.02 (1.01–1.04)*	1.03 (1.03–1.04)*
Mediators (%)				
Depressive symptoms			16.92	18.57
Social isolation				
Increasing multisite vs minimal multisite (multinomial logit)				
Total effect^b^	1.10 (0.92–1.22)	1.18 (1.06–1.34)*	1.38 (1.35–1.70)*	1.50 (1.27–1.69)*
Direct effect	1.08 (0.98–1.15)	1.14 (1.02–1.29)*	1.29 (1.10–1.38)*	1.36 (1.13–1.48)*
Indirect effect	1.02 (1.01–1.04)*	1.04 (1.03–1.06)*	1.07 (1.05–1.10)*	1.10 (1.08–1.13)*
Mediators (%)				
Depressive symptoms	15.15	25.45	20.63	24.50
Social isolation				
High multisite vs minimal multisite (multinomial logit)				
Total effect^c^	1.26 (1.13–1.47)*	1.45 (1.28–1.71)*	1.89 (1.59–2.30)*	2.50 (2.36–2.84)*
Direct effect	1.19 (1.05–1.41)*	1.29 (1.13–1.48)*	1.57 (1.31–1.97)*	1.92 (1.64–2.27)*
Indirect effect	1.06 (1.03–1.11)*	1.13 (1.09–1.16)*	1.23 (1.19–1.27)*	1.37 (1.32–1.43)*
Mediators (%)				
Depressive symptoms	17.98	28.94	26.61	28.25
Social isolation	1.75	1.34	1.73	1.53

“a” represents a Cox model, with hazard ratios (HR) presented for total, direct, and indirect effects on the association between ACEs and pain incidence risk.

“b and c” represent the multinomial model, with odds ratios (OR) presented for total, direct, and indirect effects on the association between ACEs and pain trajectories. Specifically, “b” assesses mediation in the comparison of increasing multisite vs minimal multisite, whereas “c” assesses mediation in the comparison of high multisite vs minimal multisite.

Estimates are based on the MMA (Multiple Mediation Analysis) method. Percentages (%) indicate the relative contributions of each mediator to the indirect effect. Contributions represent the portion of the indirect effect attributable to each mediator and are only displayed when the indirect effect of the mediator is significant. ACEs were derived from 13 items, each dichotomized and summed to create an ACEs score, with higher scores indicating greater adversity levels. Both models are adjusted for age, sex, marital status, region, education, smoking, drinking, annual per capita household expenditure, and chronic diseases.

* 95% CI excludes 1.00 (2-sided *P* < 0.05).

ACEs, adverse childhood experiences; CI, confidence interval; HR, hazard ratio; OR, odds ratio.

In the Cox proportional hazards model evaluating pain incidence, the total effect of ACEs on pain incidence for participants with an ACEs score of 4 or more was significant (HR = 1.21; 95% CI: 1.14–1.25). Of the 2 tested mediators, the indirect effect through depressive symptoms was significant (HR = 1.03; 95% CI: 1.03–1.04), accounting for 18.57% of the total effect. The indirect effect through social isolation was not statistically significant. The direct effect of ACEs on pain incidence, after accounting for mediators, remained significant (HR = 1.17; 95% CI: 1.11–1.21), suggesting partial mediation primarily driven by depressive symptoms.

In the multinomial logistic regression model evaluating multisite pain trajectory groups, the total effect of ACEs on the increasing multisite trajectory group (compared to the minimal multisite trajectory) was significant (OR = 1.50; 95% CI: 1.27–1.69) for participants with an ACEs score of 4 or more. Of the 2 tested mediators, depressive symptoms mediated 24.50% of the indirect effect, with an OR of 1.10 (95% CI: 1.08–1.13). Social isolation's contribution to the mediation was negligible and not statistically significant. For the high multisite trajectory group (compared to the minimal multisite trajectory), the total effect was significant (OR = 2.50; 95% CI: 2.36–2.84). Depressive symptoms mediated 28.25% of the effect (indirect effect OR = 1.10; 95% CI: 1.08–1.13), whereas social isolation accounted for 1.53% of the effect (indirect effect OR = 1.02; 95% CI: 1.01–1.04). By contrast, social isolation contributed minimally and was not statistically significant in most models. These findings underscore the central role of depressive symptoms in linking ACEs to both the onset and progression of multisite pain distribution over time.

## 4. Discussion

This study examined the association between ACEs and pain outcomes, including incidence and trajectories in the number of painful body sites (multisite pain), among middle-aged and older adults in China across a 9-year period. The results show that higher ACEs scores are associated with an increased risk of pain incidence and multisite pain trajectories. Of the 2 tested mediators, depressive symptoms explained a larger share of the ACEs–pain association, whereas mediation via social isolation was relatively weak. These findings align with broader evidence that early adversity shapes later health outcomes and underscore the significant public health burden posed by pain in middle-aged to older adults. By investigating the mediating role of depression and social isolation, this study contributes new insights into the psychosocial mechanisms that link ACEs to pain outcomes.

The observed association between ACEs and pain outcomes aligns with existing research conducted in other populations.^4,26^ Among the Chinese population, a cross-sectional study demonstrated that ACEs are associated with an increased risk of experiencing pain, such as neck, shoulder, and back pain, with a dose–response relationship observed.^24^ Furthermore, a longitudinal study also showed that ACEs are associated with the risk of pain and its trajectory over time, underscoring the long-term effects of early adversities on chronic pain progression.^23^ This study further substantiates existing evidence, indicating that higher ACEs scores are significantly associated with an increased risk of pain onset and the progression of multisite pain trajectories over time. By employing a longitudinal design, this study contributes to the literature by providing comprehensive estimates of the relationship between ACEs and pain while investigating the mediation effects of psychological and social factors.

This study highlights the critical mediating role of depressive symptoms in the association between ACEs and pain outcomes. Depressive symptoms accounted for a significant proportion of the indirect effect, particularly for individuals after the high multisite pain trajectory. Previous studies also found that depression mediates the relationship between ACEs and chronic lung diseases,^20^ asthma,^37^ and cardiovascular disease,^6^ demonstrating that depression exerts a strong mediation effect on the progression and severity of these conditions. This evidence supports the hypothesis that depression is one pathway linking childhood adversity to pain. Depression may amplify pain perception, impair coping mechanisms, and intensify emotional distress, which contributes to the persistence and development of pain.^21^ Addressing depression through interventions such as cognitive–behavioral therapy or pharmacological treatments could mitigate the impact of ACEs on chronic pain outcomes^10^ as part of multicomponent, trauma-informed approaches. Nonetheless, depression is not the sole pathway; other psychosocial mechanisms like catastrophizing, sleep disturbance, anxiety/Post-Traumatic Stress Disorder (PTSD), and loneliness may also likely contribute and warrant evaluation.

The potential mechanisms of depression on pain development are complex and involve multiple factors. The cognitive–behavioral model suggests that depression leads to catastrophizing, where individuals tend to exaggerate the intensity and long-term consequences of pain. This type of thinking not only worsens the perception of pain but also increases emotional distress, thereby amplifying the pain experience.^9^ According to the biopsychosocial model, depression affects both psychological and biological factors that influence pain. Depression has been linked to central sensitization, which lowers the threshold for pain perception, making pain feel more intense.^32^ In addition, depression is associated with increased inflammation, which can exacerbate pain, particularly in rheumatoid arthritis.^17^

In contrast, social isolation was also identified as a mediator; however, its effect was relatively weak and observed only in the high multisite trajectory group, compared to the stronger contribution of depressive symptoms. This finding is consistent with the understanding that social relationships play a protective role in health by buffering stress and providing emotional and practical support.^38^ Although the role of social isolation was less pronounced, addressing social connectivity in older adults, particularly those with high ACEs exposure, could still have beneficial effects in reducing pain burden.^7^

### 4.1. Strengths and limitations

This study has several strengths. First, we used a nationally representative dataset, ensuring that the findings are applicable to the Chinese population and increasing external validity. Second, the longitudinal design enabled estimation of incident pain and multisite pain trajectories across 2011 to 2020. Third, we examined psychosocial mediating pathways through which ACEs may influence pain, adding to the literature by identifying potential intervention points that could help mitigate the impact of ACEs on long-term health. However, there are some limitations. First, ACEs and pain were self-reported; outcomes index the number of painful sites (distribution) rather than intensity or phenotype; and the social isolation composite is not a validated scale and does not capture loneliness/relationship quality. However, previous studies have found that the recall bias of ACEs does not invalidate its measurement.^13^ These issues should be addressed in future work using validated loneliness and anxiety scales and phenotype-specific pain outcomes. Second, the observational design limits causal inference; attrition and missingness could bias estimates—we used multiple imputation under an MAR assumption and results were similar in sensitivity analyses; some trajectory class misclassification is possible, although diagnostics indicated acceptable separation. Third, this study focuses on the Chinese population may limit its generalizability to other cultural contexts, where perceptions of pain and emotional expression differ.

## 5. Conclusion

In conclusion, ACEs were associated with increased risk of body pain incidence and multisite pain trajectories. Among the 2 parallel mediators examined, depressive symptoms explained a larger share of the indirect effect than social isolation. Focusing on depression through psychological or pharmacological interventions may reduce the pain burden in older adults with high ACEs. Future research should continue to investigate additional mediators and explore the effectiveness of targeted interventions, especially in diverse cultural contexts, to better address the psychosocial components of pain management.

## Disclosures

The authors have no conflict of interest to declare.

## Supplemental digital content

Supplemental digital content associated with this article can be found online at http://links.lww.com/PR9/A324.

## Supplementary Material
