# Supplementary material for: Adverse childhood experiences and pain incidence and multisite trajectories among middle-aged and older Chinese adults
Source: Pain Rep. 2026 Jan 9;11(1):e1389. doi: 10.1097/PR9.0000000000001389 (PMC13630268; doi:10.1097/PR9.0000000000001389)
Supplement: Supplementary file 1 [file painreports-11-e1389-s001.pdf]

Supplementary Materials — Contents

Manuscript title: Adverse Childhood Experiences and Pain Incidence and Multisite Trajectories among Middle-Aged and Older Chinese Adults

Authors: Jiajia Li, Heming Pei, Guilan Xie, Gong Chen, Lijun Pei

This file includes:

Figures

Supplementary Figure S1. Conceptual mediation framework for ACEs and pain outcomes

Supplementary Figure S2. Missing-data heatmap for control variables—incidence (Cox) cohort (N = 9,252)

Supplementary Figure S3. Missing-data heatmap for control variables—trajectory cohort (5-wave sample) (N = 9,935)

Tables

Supplementary Table S1. Social isolation items and coding criteria

Supplementary Table S2. Model selection criteria and incremental improvement for group-based trajectory modeling (multisite pain)

Supplementary Table S3. Missingness summary and Little's MCAR test—incidence (Cox) cohort

Supplementary Table S4. Missingness summary and Little's MCAR test—trajectory cohort (5-wave sample)

Supplementary Table S5. Baseline characteristics by follow-up status—incidence (Cox) cohort (N = 9,252)

Supplementary Table S6. Baseline characteristics by trajectory data completeness—trajectory cohort (baseline N = 13,907; complete 5-wave N = 9,935)

Supplementary Table S7. Inverse-probability-of-attrition–weighted (IPW) estimates for ACEs and pain outcomes

Figure S1: Conceptual Mediation Framework for ACEs and Pain Outcomes

Common pathways across different pain outcome models

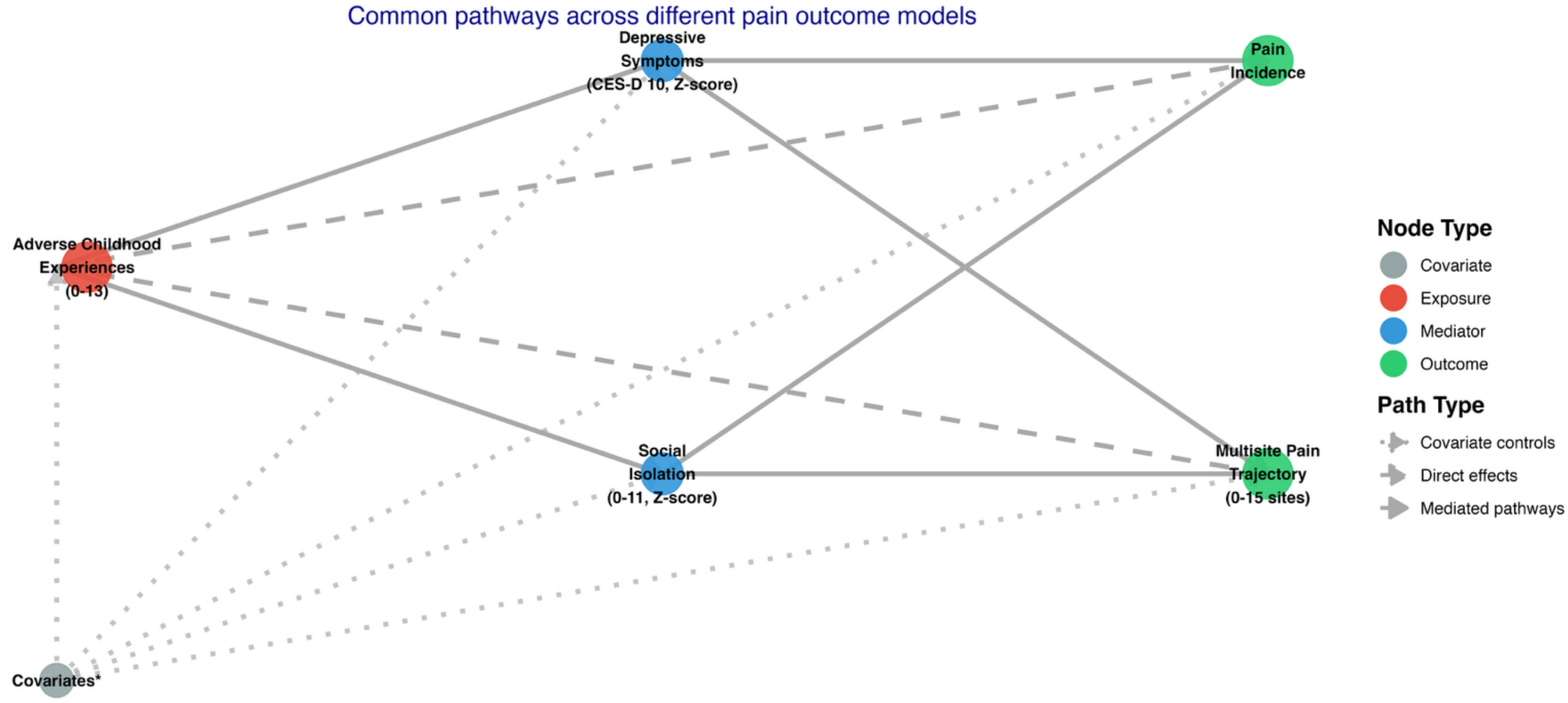

*Covariates: Age, sex, education, marital status, residence, smoking, drinking, income, chronic diseases.
**Pain Incidence: Cox proportional hazards model (time-to-event)
**Pain Trajectory: Multinomial logistic regression (trajectory groups)
Mediator timing: Lagged (T+1) for incidence analysis; baseline for trajectory analysis.
Dashed lines = direct effects; Solid lines = mediated pathways; Dotted lines = covariate controls.

Supplementary Figure S1. Conceptual mediation framework for ACEs and pain outcomes

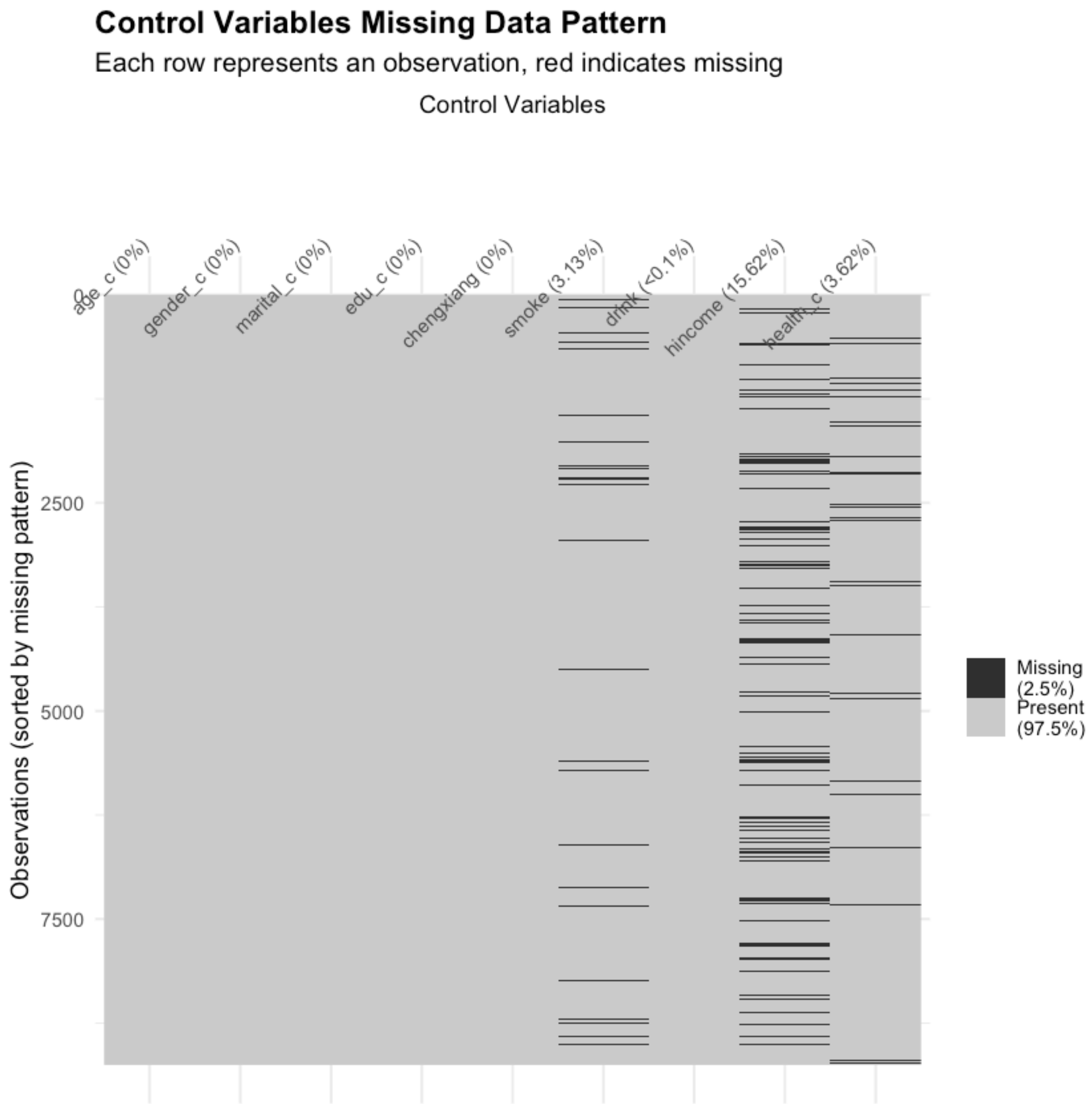

Supplementary Figure S2. Missing-data heatmap for control variables—incidence (Cox) cohort (N=9,252).

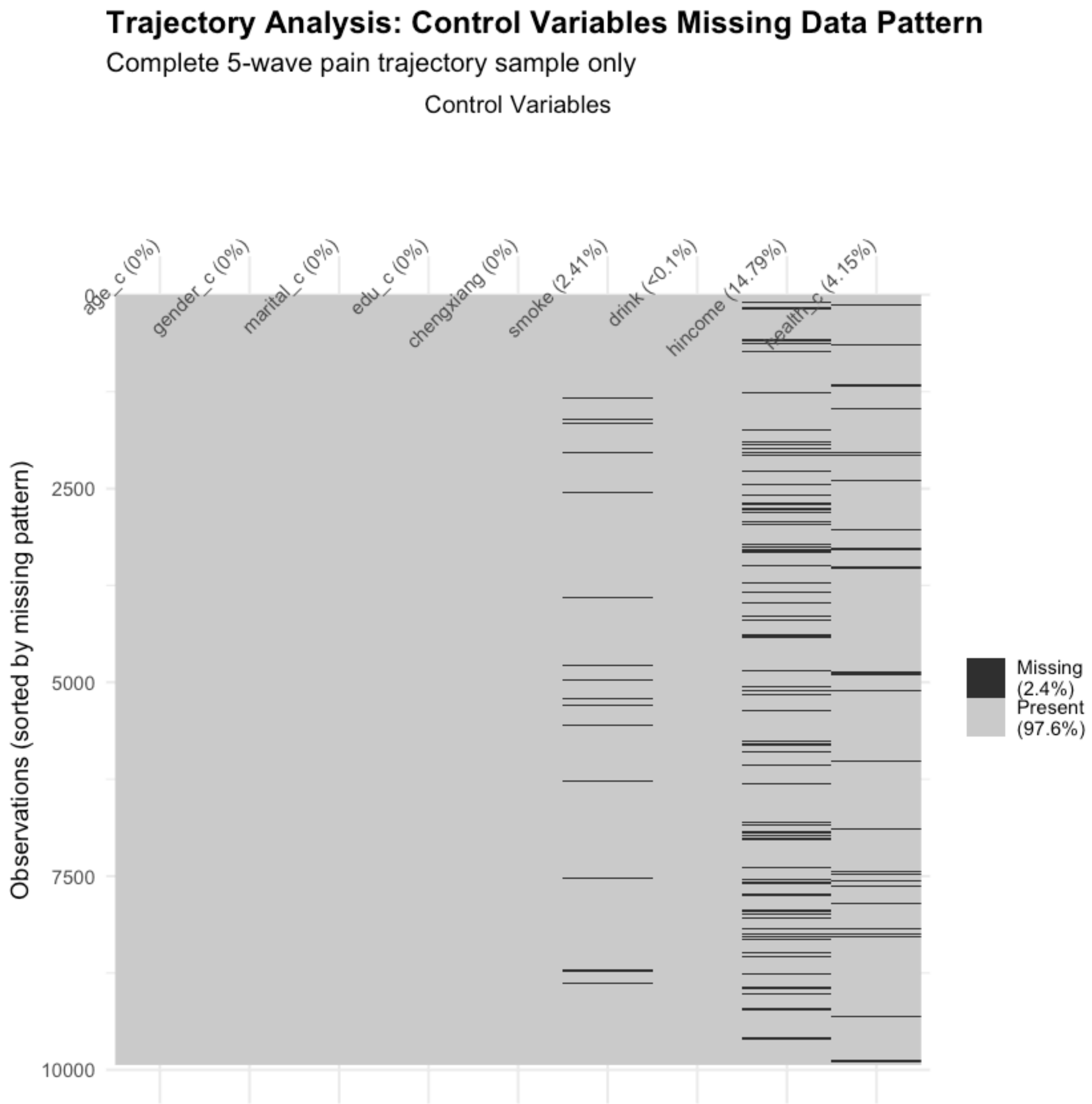

Supplementary Figure S3. Missing-data heatmap for control variables—trajectory cohort (5-wave sample) (N=9,935).

Supplementary Table S1. Social isolation items and coding criteria

Item / question (as asked)	Definition criteria (Isolation = 1, Engagement = 0)
Ever married by the current wave	**1** = Never married; **0** = Ever married
Any children co-reside with the respondent	**1** = No child co-resides; **0** = ≥1 child co-resides
Any children live in the same city/county as the respondent	**1** = No child lives nearby; **0** = ≥1 child lives nearby
Any weekly in-person contact with parents/parents-in-law	**1** = No weekly in-person contact; **0** = Any weekly in-person contact
Any weekly in-person contact with children	**1** = No weekly in-person contact; **0** = Any weekly in-person contact
Interacted with friends	**1** = Did **not** do in the last month; **0** = Did
Played Ma-jong/chess/cards or went to community club	**1** = Did **not** do in the last month; **0** = Did
Went to a sport, social, or other kind of club	**1** = Did **not** do in the last month; **0** = Did
Took part in a community-related organization	**1** = Did **not** do in the last month; **0** = Did
Done voluntary or charity work	**1** = Did **not** do in the last month; **0** = Did
Cared for a sick/disabled non-cohabiting adult without pay	**1** = Did **not** do in the last month; **0** = Did

Note: Recode each item so 1 = more isolated and 0 = more engaged; sum across all 11 items to form the raw index (range 0–11; higher = greater social isolation). For comparability across waves, compute a wave-specific z-score. Document how structurally inapplicable cases are handled (e.g., no living parents/parents-in-law; no children): either code as 1 to retain the 11-item scale, or set to missing and prorate in sensitivity analyses.

Supplementary Table S2. Model Selection Criteria and Incremental Improvement for Group-Based Trajectory Modeling (Multisite Pain)

Groups	Log-Likelihood	AIC[1]	BIC[1]	CVE[2]	ΔBIC[3]	%ΔBIC	ΔCVE[3]	%ΔCVE
2	−85,060.23	170,142.46	170,239.40	1.7432	–	–	–	–
3	−81,592.11	163,230.21	163,432.92	1.4905	−6,806.48	−4.0%	−0.2527	−14.5%
4	−81,862.21	163,758.42	163,908.24	1.4994	+475.32	+0.3%	+0.0089	+0.6%
5	−81,052.09	162,162.17	162,417.76	1.4744	−1,490.48	−0.9%	−0.0250	−1.7%

[1] Lower AIC and BIC values indicate better balance of fit and parsimony.

[2] CVE = Cross-Validation Error; lower values indicate better predictive accuracy.

[3] Δ statistics represent change from the previous model (e.g., 3-group vs. 2-group)

Supplementary Table S3. Missingness summary and Little's MCAR test—incidence (Cox) cohort

Little's MCAR Test: chi-squared = 783, df = 72, p = 0

Control Variables	Complete Observations	Missing Count	Missing Percentage (%)[1]
Household income quartile	7,807	1,445	15.62
Number of chronic diseases	8,917	335	3.62
Smoking status	8,962	290	3.13
Drinking status	9,246	6	0.06
Age group	9,252	0	0
Gender	9,252	0	0
Marital status	9,252	0	0
Education level	9,252	0	0
Rural/Urban residence	9,252	0	0

[1] Red highlighting indicates missing percentage > 5%

Supplementary Table S4. Missingness summary and Little's MCAR test—trajectory cohort (5-wave sample)[1]

Complete 5-wave trajectory sample (N=9935); Little's MCAR Test: chi-squared = 679.89, df = 76, p = 0[1]

Control Variables	Complete Observations	Missing Count	Missing Percentage (%)[2]
Household income quartile	8,466	1,469	14.79
Number of chronic diseases	9,523	412	4.15
Smoking status	9,696	239	2.41
Drinking status	9,926	9	0.09
Age group	9,935	0	0
Gender	9,935	0	0
Marital status	9,935	0	0
Education level	9,935	0	0
Rural/Urban residence	9,935	0	0

[1] Analysis limited to participants with complete pain trajectory data across all 5 waves (2011-2020)

[2] Red highlighting indicates missing percentage > 5%

Supplementary Table S5. Baseline characteristics by follow-up status—incidence (Cox) cohort (N=9,252) [1]

Baseline Characteristics	Comparison across different follow-up outcomes[1]				p-value[3, 4]
	Overall	Right Cencor	Lost to Follow-up	Pain Developed	
	N = 9252[2]	N = 1544[2]	N = 1377[2]	N = 6331[2]	
**Adverse Childhood Experiences**					<0.001
0	1,138 (12.3%)	205 (13.3%)	250 (18.2%)	683 (10.8%)	
1	2,136 (23.1%)	408 (26.4%)	343 (24.9%)	1,385 (21.9%)	
2	2,357 (25.5%)	438 (28.4%)	353 (25.6%)	1,566 (24.7%)	
3	1,727 (18.7%)	255 (16.5%)	210 (15.3%)	1,262 (19.9%)	
≥4	1,894 (20.5%)	238 (15.4%)	221 (16.0%)	1,435 (22.7%)	
**Age group (years)**					<0.001
45-49	1,997 (21.6%)	359 (23.3%)	259 (18.8%)	1,379 (21.8%)	
50-54	1,456 (15.7%)	261 (16.9%)	177 (12.9%)	1,018 (16.1%)	
55-59	1,972 (21.3%)	364 (23.6%)	234 (17.0%)	1,374 (21.7%)	
60-64	1,528 (16.5%)	264 (17.1%)	199 (14.5%)	1,065 (16.8%)	
65-69	975 (10.5%)	161 (10.4%)	139 (10.1%)	675 (10.7%)	
70-74	690 (7.5%)	86 (5.6%)	162 (11.8%)	442 (7.0%)	
≥75	634 (6.9%)	49 (3.2%)	207 (15.0%)	378 (6.0%)	
**Gender**					<0.001
Female	4,352 (47.0%)	555 (35.9%)	495 (35.9%)	3,302 (52.2%)	
Male	4,900 (53.0%)	989 (64.1%)	882 (64.1%)	3,029 (47.8%)	
**Marital status**					<0.001
Married	7,625 (82.4%)	1,360 (88.1%)	1,036 (75.2%)	5,229 (82.6%)	
Not married	1,627 (17.6%)	184 (11.9%)	341 (24.8%)	1,102 (17.4%)	
**Education level**					<0.001
College and above	1,288 (13.9%)	268 (17.4%)	237 (17.2%)	783 (12.4%)	
High school	2,094 (22.6%)	409 (26.5%)	314 (22.8%)	1,371 (21.7%)	
Middle school	2,015 (21.8%)	333 (21.6%)	290 (21.1%)	1,392 (22.0%)	
No formal education	2,280 (24.6%)	283 (18.3%)	321 (23.3%)	1,676 (26.5%)	
Primary school	1,575 (17.0%)	251 (16.3%)	215 (15.6%)	1,109 (17.5%)	
**Residence**					<0.001
Rural	5,515 (59.6%)	920 (59.6%)	666 (48.4%)	3,929 (62.1%)	
Urban	3,737 (40.4%)	624 (40.4%)	711 (51.6%)	2,402 (37.9%)	
**Smoking status**					<0.001
Current smoker	2,858 (30.9%)	568 (36.8%)	471 (34.2%)	1,819 (28.7%)	

Missing	290 (3.1%)	39 (2.5%)	82 (6.0%)	169 (2.7%)	
Non-smoker	6,104 (66.0%)	937 (60.7%)	824 (59.8%)	4,343 (68.6%)	
**Drinking status**					<0.001
Current drinker	3,318 (35.9%)	644 (41.7%)	527 (38.3%)	2,147 (33.9%)	
Missing	6 (0.1%)	0 (0.0%)	2 (0.1%)	4 (0.1%)	
Non-drinker	5,928 (64.1%)	900 (58.3%)	848 (61.6%)	4,180 (66.0%)	
**Household income quartile**					<0.001
Missing	1,445 (15.6%)	212 (13.7%)	249 (18.1%)	984 (15.5%)	
Q1 (Lowest)	1,938 (20.9%)	322 (20.9%)	279 (20.3%)	1,337 (21.1%)	
Q2	1,930 (20.9%)	303 (19.6%)	241 (17.5%)	1,386 (21.9%)	
Q3	2,006 (21.7%)	359 (23.3%)	296 (21.5%)	1,351 (21.3%)	
Q4 (Highest)	1,933 (20.9%)	348 (22.5%)	312 (22.7%)	1,273 (20.1%)	
**Number of chronic diseases**					<0.001
0	3,444 (37.2%)	807 (52.3%)	544 (39.5%)	2,093 (33.1%)	
1	2,781 (30.1%)	450 (29.1%)	389 (28.2%)	1,942 (30.7%)	
2	1,599 (17.3%)	158 (10.2%)	230 (16.7%)	1,211 (19.1%)	
3	649 (7.0%)	59 (3.8%)	100 (7.3%)	490 (7.7%)	
≥4	444 (4.8%)	18 (1.2%)	60 (4.4%)	366 (5.8%)	
Missing	335 (3.6%)	52 (3.4%)	54 (3.9%)	229 (3.6%)	

[1] Pain developed: Participants who developed pain during follow-up; Completed follow-up: Participants who completed all waves without developing pain; Lost to follow-up: Participants who dropped out before study completion

[2] Mean (SD) for continuous variables; N (%) for categorical variables

[3] Pearson's Chi-squared test

[4] P-values from ANOVA for continuous variables and chi-square tests for categorical variables

Supplementary Table S6. Baseline characteristics by trajectory data completeness—trajectory cohort (baseline N=13,907; complete 5-wave N=9,935)

Comparison between participants with complete vs. incomplete trajectory data

Baseline Characteristics	Overall	Complete Trajectory Data	Incomplete Trajectory Data	p-value[2,3]
	N = 13907[1]	N = 9935[1]	N = 3972[1]	
**Adverse Childhood Experiences**				<0.001
0	1,553 (11.2%)	978 (9.8%)	575 (14.5%)	
1	2,936 (21.1%)	2,065 (20.8%)	871 (21.9%)	
2	3,418 (24.6%)	2,478 (24.9%)	940 (23.7%)	
3	2,681 (19.3%)	1,969 (19.8%)	712 (17.9%)	
≥4	3,319 (23.9%)	2,445 (24.6%)	874 (22.0%)	
**Age group**				<0.001
45-49	2,797 (20.1%)	2,112 (21.3%)	685 (17.2%)	
50-54	2,183 (15.7%)	1,636 (16.5%)	547 (13.8%)	
55-59	2,992 (21.5%)	2,281 (23.0%)	711 (17.9%)	
60-64	2,365 (17.0%)	1,802 (18.1%)	563 (14.2%)	
65-69	1,551 (11.2%)	1,083 (10.9%)	468 (11.8%)	
70-74	1,051 (7.6%)	615 (6.2%)	436 (11.0%)	
≥75	968 (7.0%)	406 (4.1%)	562 (14.1%)	
**Gender**				<0.001
Female	7,208 (51.8%)	5,319 (53.5%)	1,889 (47.6%)	
Male	6,699 (48.2%)	4,616 (46.5%)	2,083 (52.4%)	
**Marital status**				<0.001
Married	11,336 (81.5%)	8,389 (84.4%)	2,947 (74.2%)	
Not married	2,571 (18.5%)	1,546 (15.6%)	1,025 (25.8%)	
**Education level**				0.001
College and above	1,584 (11.4%)	1,066 (10.7%)	518 (13.0%)	
High school	2,837 (20.4%)	2,064 (20.8%)	773 (19.5%)	
Middle school	2,974 (21.4%)	2,160 (21.7%)	814 (20.5%)	
No formal education	3,913 (28.1%)	2,778 (28.0%)	1,135 (28.6%)	
Primary school	2,599 (18.7%)	1,867 (18.8%)	732 (18.4%)	
**Residence**				<0.001
Rural	8,807 (63.3%)	6,627 (66.7%)	2,180 (54.9%)	
Urban	5,100 (36.7%)	3,308 (33.3%)	1,792 (45.1%)	
**Smoking status**				<0.001
Current smoker	4,036 (29.0%)	2,861 (28.8%)	1,175 (29.6%)	

Missing	420 (3.0%)	239 (2.4%)	181 (4.6%)	
Non-smoker	9,451 (68.0%)	6,835 (68.8%)	2,616 (65.9%)	
**Drinking status**				0.009
Current drinker	4,641 (33.4%)	3,291 (33.1%)	1,350 (34.0%)	
Missing	21 (0.2%)	9 (0.1%)	12 (0.3%)	
Non-drinker	9,245 (66.5%)	6,635 (66.8%)	2,610 (65.7%)	
**Household income quartile**				0.002
Q1	3,033 (25.7%)	2,157 (25.5%)	876 (26.4%)	
Q2	2,982 (25.3%)	2,192 (25.9%)	790 (23.8%)	
Q3	2,983 (25.3%)	2,181 (25.8%)	802 (24.2%)	
Q4	2,784 (23.6%)	1,936 (22.9%)	848 (25.6%)	
Missing	2,125	1,469	656	
**Number of chronic diseases**				<0.001
0	4,247 (31.9%)	3,138 (33.0%)	1,109 (29.2%)	
1	4,006 (30.1%)	2,910 (30.6%)	1,096 (28.9%)	
2	2,670 (20.1%)	1,873 (19.7%)	797 (21.0%)	
3	1,336 (10.0%)	927 (9.7%)	409 (10.8%)	
≥4	1,057 (7.9%)	675 (7.1%)	382 (10.1%)	
Missing	591	412	179	

[1] Mean (SD) for continuous variables; N (%) for categorical variables

[2] Pearson's Chi-squared test; Welch Two Sample t-test

[3] P-values from t-tests for continuous variables and chi-square tests for categorical variables

Supplementary Table S7. Inverse-probability-of-attrition–weighted (IPW) estimates for ACEs and pain outcomes.

Variables	Model 1			Model 2	
ACEs score	No. of participants	No. of events	HR(95%CI)	Low-moderate V.S. Low-low OR(95%CI)	Moderate-severe V.S. Low-low OR(95%CI)
0	1,138	683	1.00	1.00	1.00
1	2,136	1,385	0.99 (0.89 ~ 1.09)	1.27 (1.05 ~ 1.54)	1.31 (0.96 ~ 1.79)
2	2,357	1,566	1.00 (0.91 ~ 1.11)	1.40 (1.16 ~ 1.69)	1.59 (1.18 ~ 2.14)
3	1,727	1,262	1.22 (1.10 ~ 1.35)	1.94 (1.60 ~ 2.35)	2.74 (2.03 ~ 3.69)
≥4	1,894	1,435	1.34 (1.20 ~ 1.48)	2.32 (1.92 ~ 2.80)	3.91 (2.92 ~ 5.24)

Note: Model 1 is a Cox proportional hazards model examining the association between ACEs and pain incidence risk, while Model 2 is a multinomial logistic model assessing the association between ACEs and pain trajectory groups. Both models are adjusted for age, sex, marital status, region, education, smoking, drinking, annual per capita household expenditure level, and chronic pain. Hazard ratios (HR) with 95% confidence intervals (CI) are presented for Model 1, while odds ratios (OR) with 95% CI are presented for Model 2.

Abbreviations: ACEs = Adverse Childhood Experiences; HR = Hazard Ratio; OR = Odds Ratio; CI = Confidence Interval.
